# Neoadjuvant S‐1 and oxaliplatin plus bevacizumab therapy for high‐risk locally advanced rectal cancer: A prospective multicenter phase II study

**DOI:** 10.1002/ags3.12720

**Published:** 2023-07-20

**Authors:** Takuya Miura, Hajime Morohashi, Yoshiyuki Sakamoto, Takuji Kagiya, Tatsuya Hasebe, Yoshihito Nakayama, Hiromasa Fujita, Kenichi Hakamada

**Affiliations:** ^1^ Department of Gastroenterological Surgery Hirosaki University Graduate School of Medicine Hirosaki Japan; ^2^ Department of Surgery Odate Municipal General Hospital Odate Japan; ^3^ Department of Surgery Hachinohe City Hospital Hachinohe Japan; ^4^ Department of Radiology Hirosaki University Graduate School of Medicine Hirosaki Japan

**Keywords:** bevacizumab, locally advanced rectal cancer, neoadjuvant chemotherapy, oxaliplatin, S‐1

## Abstract

**Aim:**

We report the short/mid‐term results of surgery for high‐risk locally advanced rectal cancer (LARC) after neoadjuvant chemotherapy (NAC, four courses of S‐1 + oxaliplatin+ bevacizumab) without radiotherapy with the primary aim of ypT0‐2.

**Methods:**

High‐risk LARC was defined as cT4b, mesorectal fascia (MRF) ≤1 mm (MRF+), or lateral lymph node metastasis (cLLN+) on high‐resolution MRI. The planned 32 cases from April 2018 to December 2021 were all included.

**Results:**

There were 10 patients at cT4b (31.2%), 26 MRF+ (81.3%), and 22 cLLN+ (68.8%). Thirteen (40.6%) underwent NAC after a colostomy for stenosis. NAC was completed in 26 (81.2%) cases. Grade 3 or higher adverse events occurred in six (18.7%). One patient developed progressive disease (3.2%). Eleven were ycT0‐3MRF‐LLN‐ (34.3%). Curative‐intent surgery was performed on 31, with sphincter‐preserving surgery in 20, abdominoperineal resection in nine, total pelvic exenteration in two, and lateral lymph node dissection in 24. Two had R1/2 resection (6.4%). A Grade 3 or higher postoperative complication rate occurred in 3.2%. Pathological complete response and ypT0‐2 rates were 12.9% and 45.1%. Three‐year disease‐free survival rates (3yDFS) for ypT0‐2 and ypT ≥3 were 81.2%, 46.6% (*p* = 0.061), and 3‐year local recurrence rates (3yLR) were 0%, 48.8% (*p* = 0.015). 3yDFS for ycT0‐3MRF‐LLN‐ and ycT4/MRF+/LLN+ were 87.5%, 48.0% (*p* = 0.031) and 3yLR were 0%, 42.8% (*p* = 0.045).

**Conclusion:**

NAC yielded a clinically significant effect in about half of high‐risk LARC patients. If NAC alone is ineffective, radiotherapy should be added, even if extended surgery is intended.

## Graphical abstract

We report the short/mid‐term results of surgery for high‐risk locally advanced rectal cancer (LARC) after neoadjuvant chemotherapy (NAC, four courses of S‐1 + oxaliplatin+ bevacizumab) without radiotherapy with the primary aim of ypT0‐2. NAC yielded a clinically significant effect in about half of high‐risk LARC patients.

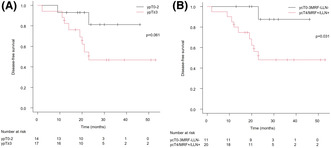

## 1 INTRODUCTION

Although curative resection of localized rectal cancer is achieved with the standard procedure of autonomic nerve‐preserving total mesorectal excision (TME), extended surgical treatment is a strategy for high‐risk locally advanced rectal cancer (LARC) that is unresectable with TME. However, it has been shown that extended surgery alone results in a high rate of recurrence, with about half of affected patients dying of cancer.^1^, ^2^ Preoperative radiotherapy (RT) has been the main approach for LARC around the world, but its ineffectiveness in reducing systemic recurrence and radiation‐induced late toxicity has led to the desire for the development of effective radiation‐independent treatments.^3^, ^4^

In this context, it has been reported that multi‐agent chemotherapy and molecular‐targeted agents for unresectable colorectal cancer are effective not only for metastatic disease but also for primary lesions, and that radiotherapy may be avoided in LARC.^5^, ^6^ On the other hand, in past reports, the pathologic response rate (pCR) with neoadjuvant chemotherapy (NAC) alone was not high (4.3%–13.3%) for high‐risk LARC, with bevacizumab contributing to a high incidence of anastomotic leakage.^5^, ^6^ However, it has been shown that a high cure rate was achieved if it were reduced to an intramural lesion (ypT0‐2).^7^ Therefore, with surgery as a prerequisite, it is considered sufficient to obtain ypT0‐2 in preoperative treatment. In addition, NAC for LARC can still be considered because it is presumed to be safe if a diverting stoma is created in anastomotic cases. Thus, we conducted this study to evaluate the safety and efficacy of S‐1 + oxaliplatin+bevacizumab (SOX+Bmab) in patients with high‐risk LARC based on the diagnosis from high‐resolution MRI, with the goal of ypT0‐2 and diverting stoma creation in all anastomotic procedures. We report here the results of this study.

## 2 PATIENTS AND METHODS

### 2.1 Patients

This was a single‐arm, multi‐institutional, prospective phase II study evaluating the safety and efficacy of NAC followed by surgery for high‐risk LARC. This study was conducted with the approval of Hirosaki University General Certified Review Board and registered with the Japan Registry of Clinical Trials (jRCTs021180023). All participating hospitals also gave their consent to conduct this study. Written informed consent was obtained from each patient before enrollment.

### 2.2 Eligibility criteria

Inclusion criteria were as follows: (1) histologically confirmed adenocarcinoma; (2) clinical stage T3‐4 or N0‐2 and M0; (3) the lower edge of the tumor occurring within 12 cm of the anal verge; (4) invasion to the adjacent organs (cT4b) or mesorectal fascia from the tumor being ≤1 mm (MRF+) or lateral lymph node metastasis (cLLN+) as determined by high resolution MRI; (5) an age of 18–75 years; (6) no prior anti‐tumor therapy (radiation therapy, chemotherapy or hormone therapy); (7) adequate function of important organs; a leukocyte count of 3000–12 000/mm^3^, a neutrophil count of ≥1500/mm^3^, a platelet count of ≥100 000/mm^3^, a hemoglobin concentration of ≥9.0 g/dL, a serum total bilirubin level of ≤2.0 mg/dL, aspartate aminotransferase and alanine aminotransferase levels of <100 IU/L, a serum creatinine level of ≤1.2 mg/dL, a creatinine clearance level of ≥60ml/min, a urine protein level of ≤1+, a prothrombin time‐international normalized ratio level of ≤1.5; (8) an electrocardiogram without clinically problematic abnormalities within 28 days of registration; (9) a score of 0–1 in Eastern Cooperative Oncology Group performance status (PS); (10) willingness on the patient's part to provide written informed consent.

Exclusion criteria was as follows: (1) gastrointestinal ulceration or bleeding; (2) sensory neuropathy; (3) severe diarrhea; (4) previous serious drug allergies; (5) pleural effusion or ascites that require therapy; (6) surgery within 28 days of registration (stoma construction was allowed); (7) thromboembolism, cerebral infarction, pulmonary infarction, or interstitial pneumonia; (8) serious complications (e.g. interstitial pneumonia, pulmonary fibrosis, kidney injury, hepatic failure, uncontrolled diabetes mellitus, uncontrolled hypertension); (9) active double cancer; (10) active infectious disease; (11) a significantly abnormal electrocardiogram or cardiovascular disease (e.g., heart failure, myocardial infarction, angina pectoris); (12) receiving flucytosine; (13) pregnant or lactating, women still capable of becoming pregnant, especially those intending to get pregnant or men who still want to father children; (14) hemoptysis; (15) gastrointestinal perforation within the past 6 months; (16) history of systemically administered steroids; (17) contraindications to S‐1, oxaliplatin, or bevacizumab; (18) HBsAg‐positive; (19) a physician has determined that the patient is ineligible for study participation due to safety considerations.

### 2.3 Neoadjuvant chemotherapy

S‐1 was administered orally at 80 mg/m^2^/day for 14 consecutive days followed by a 7‐day rest. Oxaliplatin was started intravenously on day 1, at a dose of 130 mg/m^2^/day. Bevacizumab was given intravenously also beginning on day 1, at a dose of 7.5 mg/kg/day. Twenty‐one days were assumed to be one course, and chemotherapy consisted of four courses. Surgery was carried out in 8 to 12 weeks after the end of chemotherapy. Toxicity was evaluated according to CTCAE (Common Terminology Criteria for Adverse Events), version 4.0. In case of persistent Grade 2 or higher adverse events, cytotoxic drugs were reduced to 60 mg/m^2^/day for S‐1 and oxaliplatin to a dose of 100 mg/m^2^/day.

### 2.4 Evaluation of staging and treatment effects

Digital examination, colonoscopy, biopsy, CT, and MRI were used to evaluate local extension and metastasis of rectal cancer. High‐resolution MRI was imaged and evaluated according to the Mercury study group.^8^ The evaluation of MRI was unified based on the judgment of a single radiologist. The preoperative MRI assessed tumor location, tumor height, T‐stage, N‐stage, MRF involvement, and extramural vascular invasion (EMVI).^9^ Perirectal and lateral lymph node metastasis was defined as a short axis diameter ≥5 mm.^10^ MRI after treatment was evaluated within 2 weeks prior to surgery and the same items as the pretreatment evaluation were reported in addition to MRI tumor regression grade (mrTRG).^11^ Objective response of the primary tumor was assessed by longitudinal length on the basis of the Response Evaluation Criteria in Solid Tumors (RECIST), version1.1.^12^

### 2.5 Surgery

Surgery was scheduled 8 to 12 weeks after the end of NAC and within 2 weeks after MRI evaluation. As a general surgical approach, the inferior mesenteric artery, the left colonic artery, and the inferior mesenteric vein were dissected, followed by TME. When the lower edge of a tumor was below the peritoneal reflection (i.e., lower rectal cancer), lateral lymph node dissection (LLND) was performed bilaterally according to Japanese guidelines.^13^ Bilateral LLND was also scheduled if the pretreatment MRI showed metastasis in the lateral lymph nodes (LLN) regardless of tumor location. When the prostate or uterus and vagina was judged to have invasion or broad contact after the posttreatment MRI, pelvic exenteration was indicated. An ileal conduit was created by cooperating urologists. Postoperative complications were defined according to the Clavien–Dindo classification.^14^

### 2.6 Pathological assessment

Specimens were opened longitudinally along the long axis of the rectum and immersed in formalin for at least 24 h. Tissue cutting was made at 5‐mm intervals in the direction of the short axis of the rectum. The mesorectum was not dissected from the lesion to evaluate the radial margin. Grading of specimens was not recorded. Pathologic complete response (pCR) was defined as no viable tumor cells being noted in the rectum, mesorectum, or harvested lymph nodes.

### 2.7 Adjuvant chemotherapy

The compliance of adjuvant 5‐FU + oxaliplatin therapy was as low as about 50% for rectal cancer.^15^ Therefore, S‐1 monotherapy was selected in this study as an adjuvant chemotherapy because of its efficacy, safety, and good compliance for stage II/III rectal cancer as shown by ACTS‐RC.^16^ S‐1 was administered orally at 80 mg/m^2^/day for 14 consecutive days followed by a 7‐day rest. Twenty‐one days were considered one course, and chemotherapy consisted of eight courses. Adjuvant chemotherapy was not administrated if the patient refused or the doctor judged the patient unable to tolerate it due to poor postoperative condition.

### 2.8 Endpoint and statistical analyses

The primary endpoint was the T down‐staging rate (ypT0‐2 rate). The ypT0‐2 rate was reported to be 26%–33% in high‐risk LARC after NAC.^5^, ^6^ The expected ypT0‐2 rate was set at 30% because the patients in this study were more strictly selected for LARC that was difficult to curatively resect. The number of cases was estimated to be 29 using Simon's two‐stage optimal design with a threshold ypT0‐2 rate of 10% with a two‐sided alpha error of 0.05 and a beta error of 0.2. The planned sample size was 32, considering the small number of dropouts. Secondary endpoints were the N down‐staging rate, adverse event rate during NAC, local response rate, mrTRG, surgical complications rate, R0 rate, pCR rate, local recurrence rate (LR), disease‐free survival (DFS), and overall survival (OS). LR was calculated from the date of surgery to pelvic recurrence or R2 resection, DFS was calculated from the date of enrollment to disease progression or death from any cause and OS was calculated from the date of enrollment to death from any cause, using the Kaplan–Meier method. LR and DFS were compared using a log‐rank test. Data were collected up to the time of evaluation after completion of postoperative adjuvant therapy for the last enrollee. A two‐sided *p* < 0.05 was considered statistically significant, and all statistical analyses were performed with EZR (Saitama Medical Center, Jichi Medical University, Saitama, Japan), which was a graphical user interface for R (The R Foundation for Statistical Computing, Vienna, Austria).^17^

## 3 RESULTS

### 3.1 Patient characteristics

The median age was 63 years (47–75), and 23 (71.8%) were male (Table 1). The median distance to the tumor from the anal verge was 55 (0–100) mm, and the lower edge of the tumor was below the peritoneal reflection in 28 patients (87.5%). Twenty‐five (78.2%) were cStage IIIC, 10 (31.2%) were cT4b, 26 (81.3%) were MRF+, and 22 (68.8%) were cLLN+. Thirteen patients (40.6%) were diagnosed with stenotic rectal cancer for which a decompression stoma was created.

**TABLE 1:** Patient characteristics at enrollment.

Variable	Value
Age (year)^a^	63 (47–75)
Male, *n* (%)	23 (71.8)
Body mass index (kg/m^2^)^a^	20.9 (17.3–30.1)
ECOG‐performance status	
0	31 (96.9)
1	1 (3.1)
Comorbidity, *n* (%)	10 (31.2)
Tumor size (mm)^a^	51.5 (23–91)
Distance from anal verge to tumor (mm)^a^	55 (0–100)
Lower edge of tumor below peritoneal reflection	28 (87.5)
cStage, *n* (%)	
cStage IIA	1 (3.1)
cStage IIIA	1 (3.1)
cStage IIIB	5 (15.6)
cStage IIIC	25 (78.2)
cT4b, *n* (%)	10 (31.2)
Levator ani muscle	6
Vagina or Uterus	3
Sigmoid colon or small intestine	2
MRF+, *n* (%)	26 (81.3)
cLLN positive, *n* (%)	22 (68.8)
EMVI	14 (43.8)
Tumor histology	
Well	15 (46.9)
Moderately	17 (53.1)
CEA (ng/ml)^a^	5.1 (0.8–148)
Decompression stoma	13 (40.6)

Abbreviations: CEA, Carcinoembryonic antigen; ECOG, Eastern Cooperative Oncology Groups; EMVI, Extramural venous invasion; LLN, Lateral lymph node; MRF, Mesorectal fascia.

^a^ Median (Range).

### 3.2 Neoadjuvant chemotherapy

Four courses of NAC were completed in 26 patients (81.2%) (Table 2). One patient did not complete the first course due to pelvic infection and progressive disease (PD). One patient had a circumferential tumor but was treated without a stoma as non‐stenotic because the scope could pass and colon perforation on the oral side of tumor occurred during the second course. One patient discontinued during the second course due to peripheral neuropathy, and two patients discontinued during the third course due to oxaliplatin allergy. One patient developed edema due to proteinuria during the fourth course and was not able to complete the program. The most frequent adverse event in all grades was peripheral neuropathy at 84.4%, and the incidence of Grade 3 or higher adverse events was 18.7%.

**TABLE 2:** Neoadjuvant chemotherapy.

Compliance	*N* (%)
Number of cycles	
4	26 (81.2)
3	1 (3.1)
2	2 (6.3)
1	2 (6.3)
0	1 (3.1)
Dose reductions	6 (18.8)
Non‐completion	6 (18.8)
Intrapelvic infection	1 (3.1)
Intestinal perforation	1 (3.1)
Allergy	2 (6.3)
Peripheral neuropathy	1 (3.1)
Proteinuria	1 (3.1)

Adverse events	Any grade (%)	≥G3 (%)
Leukopenia	18.8	0
Neutropenia	21.9	3.1
Anemia	65.6	0
Thrombocytopenia	18.8	3.1
AST	12.5	0
ALT	12.5	0
Fatigue	53.1	0
Appetite loss	56.3	0
Nausea	28.1	0
Diarrhea	18.8	0
Constipation	31.1	0
Stomatitis	6.3	0
Skin pigmentation	6.3	0
Hand foot syndrome	3.1	0
Peripheral neuropathy	84.4	3.1
Allergy	6.3	3.1
Nasal bleeding	37.5	0
Gastrointestinal bleeding	3.1	0
Hypertension	18.8	0
Proteinuria	6.3	0
Intrapelvic infection	3.1	3.1
Intestinal perforation	3.1	3.1

### 3.3 Evaluation of treatment effects

In the evaluation of primary rectal cancer lesions, complete response (CR) was observed in three patients (9.4%), partial response (PR) in 15 patients (46.9%), and PD in one patient (3.1%) (Table 3). Except for one PD patient and one patient who refused MRI reevaluation, mrTRG was evaluated in 30 patients, and TRG1‐2 was seen in four patients. Eight (25.0%) were ycStage IIIC, eight (25.0%) were cT4b, 18 (56.2%) were MRF+, and eight (25.0%) were cLLN+. Eleven patients (34.3%) were diagnosed with ycT0‐3MRF‐LLN‐. In these 11 cases, cT4 was observed in one, MRF+ in six, cLLN+ in seven, and EMVI in four.

**TABLE 3:** Clinical treatment effects.

Variable	Value
Objective response of primary tumor, *n* (%)	
Complete response	3 (9.4)
Partial response	15 (46.9)
Stable disease	13 (40.6)
Progressive disease	1 (3.1)
TRG	
1	2
2	2
3	13
4	11
5	2
Not assessed	2
ycStage, *n* (%)	
ycStage 0	2 (6.3)
ycStage I	2 (6.3)
ycStage IIA	7 (21.8)
ycStage IIB	2 (6.3)
ycStage IIC	4 (12.5)
ycStage IIIA	2 (6.3)
ycStage IIIB	5 (15.5)
ycStage IIIC	8 (25.0)
ycT4b, *n* (%)	8 (25.0)
yMRF+, *n* (%)	18 (56.2)
ycLLN positive, *n* (%)	8 (25.0)
yEMVI, *n* (%)	5 (15.6)
ycT0‐3MRF‐LLN‐, *n* (%)	11 (34.3)
CEA (ng/ml)^a^	2.9 (0.7–12.6)

Abbreviations: CEA, Carcinoembryonic antigen; EMVI, Extramural venous invasion; LLN, Lateral lymph node; MRF, Mesorectal fascia; TRG, Tumor regression grade.

^a^ Median (Range).

### 3.4 Surgical outcome

Curative‐intent surgery was performed in 31 cases (Table 4). Twenty‐six (83.8%) were performed as robot‐assisted, 20 (64.5%) were sphincter‐sparing, and one patient did not request reconstruction and underwent Hartmann's operation. The median distance of the anastomotic height from the anal verge was 40 (30–70) mm. Diverting stomas were created in all anastomotic cases, and stoma closure was performed in all cases except for one who did not wish to have stoma closure. Bilateral LLND was performed in 24 cases. Five cases did not undergo LLND due to colorectal perforation, patient refusal, and lower edge of the tumor above the peritoneal reflection after NAC. Two cases resulted in unilateral LLND due to technical difficulty by fibrosis after gynecologic surgery and R2 resection in the lateral region. There was only one case of a postoperative CD complication III or higher (3.2%) with anastomotic leakage. Of the 11 patients with ycT0‐3MRF‐LLN, 10 (91%) underwent sphincter‐sparing surgery.

**TABLE 4:** Surgical outcomes (*n* = 31).

Variable	Value
Approach, *n* (%)	
Robotic	26 (83.8)
Laparoscopic	3 (9.7)
Open	2 (6.5)
Type of surgery, *n* (%)	
LAR	16 (51.6)
ISR	3 (9.7)
Hartmann	1 (3.2)
APR	9 (29.0)
TPE	2 (6.5)
Anastomotic height from anal verge (mm)^a^ ^,^ ^b^	40 (30–70)
Diverting stoma, *n* (%)^b^	19 (100)
Closure of diverting stoma, *n* (%)^b^	18 (94.7)
Lateral lymph node dissection, *n* (%)	24 (77.4)
Bilateral	22
Unilateral	2
Operation time (min)^a^	470 (142–701)
Blood loss (mL)^a^	50 (0–900)
Complications (Clavien–Dindo), *n* (%)	
I‐II	21 (67.8)
III	1 (3.2)
IV‐V	0
Anastomotic leakage, *n* (%)^b^	1 (5.3)

Abbreviations: APR, Abdominoperineal resection; ISR, Intersphincteric resection; LAR, Low anterior resection; TPE, Total pelvic exenteration.

^a^ Median (Range).

^b^ LAR and ISR.

### 3.5 Pathological outcome

R0 was observed in 29 patients (93.6%) and there was a radial margin ≤1 mm in three patients (9.7%) (Table 5). Four patients (12.9%) had pCR, nine (29.0%) had pStage I, and two (6.5%) had pStage IIIC. Fourteen patients (45.1%) had ypT0‐2. Of the 30 cN+ patients, 12 were pN+ and the N down‐staging rate was 60%. Of the 22 cLLN+ patients, LLND was performed in 21, excluding one patient in which LLND was not performed due to colorectal perforation; pLLN+ was found in five patients (22.7%).

**TABLE 5:** Pathological outcomes (*n* = 31).

Variable	Value
Residual tumor classification, *n* (%)	
R0	29 (93.6)
R1	1 (3.2)
R2	1 (3.2)
Distal margin (mm)^a^	30 (5–55)
Radial margin ≤1 mm, *n* (%)	3 (9.7)
ypStage, *n* (%)	
pCR	4 (12.9)
I	9 (29.0)
IIA	6 (19.3)
IIIA	2 (6.5)
IIIB	8 (25.8)
IIIC	2 (6.5)
ypT, *n* (%)	
T0	6 (19.3)
T1	0
T2	8 (25.8)
T3	17 (54.9)
T4	0
ypN, *n* (%)	
N0	19 (61.3)
N+	12 (38.7)
LLN metastasis	5 (16.1)

Initial stage	*n*	ypT0	ypT1	ypT2	ypT3	ypT4	yp+
cT≤3	14	5	0	5	4	0	
cT4a	8	0	0	1	7	0	
cT4b	9	1	0	2	6	0	
cN+	30						12
cLLN+	22						5
EMVI+	14						10

Abbreviations: EMVI, Extramural venous invasion; LLN, Lateral lymph node; pCR, Pathological complete response.

^a^ Median (Range).

### 3.6 Oncological outcomes

**FIGURE 1:**
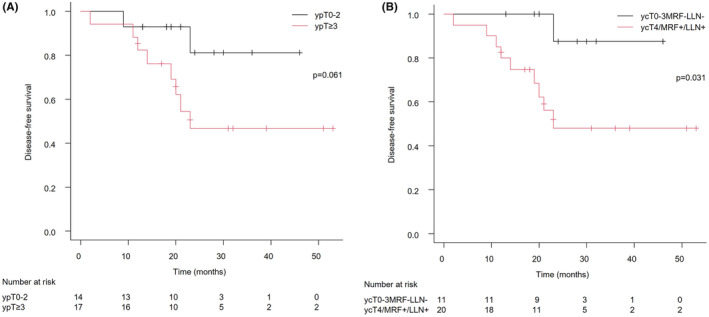
Kaplan–Meier analyses of disease‐free survival. (A) Patients with ypT0‐2 had tended to be better disease‐free survival than other patients. (B) Patients with ycT0‐3 and MRF‐ and LLN‐ was significantly better disease‐free survival than ycT4 or MRF+ or LLN+. LLN, Lateral lymph node; MRF, Mesorectal fascia.

Two patients with progressive disease or R2 resection received palliative chemotherapy. Postoperative adjuvant therapy was given to 27 patients and was completed in 26. Three patients did not receive adjuvant chemotherapy because of the patient's refusal, intestinal perforation during NAC, or postoperative complications. Overall, 3‐year OS, DFS (3yDFS), LR (3yLR) were 90.0%, 62.1%, and 27.9%, respectively. Initial recurrent organs were the lung in three patients, the liver in one patient, the peritoneum around the liver in one patient, and the pelvis in six patients. Among all local recurrences, recurrences in the central pelvis and lateral pelvis were two and four, respectively. 3yDFS for ypT0‐2 and ypT ≥3 were 81.2%, 46.6% (*p* = 0.061, Figure 1), 3yLR were 0%, 48.8% (*p* = 0.015). 3yDFS for MRF‐ and MRF+ were 100%, 51.4% (*p* = 0.061), 3yLR were 0%, 38.2% (*p* = 0.14). 3yDFS for cLLN‐ and cLLN+ were 85.7%, 50.7% (*p* = 0.062), 3yLR were 0%, 41.5% (*p* = 0.058). 3yDFS for ycT0‐3MRF‐LLN‐ and ycT4/MRF+/LLN+ were 87.5%, 48.0% (*p* = 0.031, Figure 1), 3yLR were 0%, 42.8% (*p* = 0.045).

## 4 DISCUSSION

In this study, we found that there were many cases of high‐risk LARC that could be cured by NAC followed by surgery. Several randomized control studies have demonstrated the benefit of surgery after RT for local control, and one recent randomized control trial (RCT) showed that surgery after NAC was equivalent to surgery after RT in terms of long‐term oncological outcomes.^18^ RT is known to cause bowel dysfunction and radiation‐induced late toxicity.^4^ This RCT also showed that the NAC group had significantly less postoperative bowel dysfunction than the RT group.^18^ In this way, expectations are high for NAC followed by surgery as a treatment strategy that seeks to preserve postoperative function while maintaining local control. Meanwhile, the significance of NAC has been examined as induction chemotherapy prior to RT. Among them, for the first time in recent years, a treatment strategy of induction chemotherapy followed by RT and surgery was reported to contribute to improved pCR and DFS compared to a strategy of RT followed by surgery and adjuvant chemotherapy.^19^ A study of FOLFOX+Bmab induction chemotherapy and RT followed by surgery for high‐risk LARC has also reported high pCR rates and good long‐term prognosis.^20^ Furthermore, induction chemotherapy followed by surgery and avoiding RT in good responders was verified in a tailored treatment strategy, and no local recurrence was observed in cases of significant response, suggesting that NAC is a useful treatment strategy.^21^

However, problems with multiple‐drug chemotherapy were also demonstrated, especially the bevacizumab‐related colorectal perforation observed in this study, which is an adverse event that should not be overlooked. In this study, NAC after stoma creation was stipulated for cases of stenosis, but the ability of the scope to pass through a bulky circumferential tumor meant it was not considered stenosis, and NAC was given in such a case without stoma creation. Although this study was limited to patients under 75 years of age and excluded cases with cardiovascular comorbidities based on reports of cardiovascular toxicity with oxaliplatin,^22^ some patients did not receive adequate chemotherapy due to oxaliplatin allergy or severe peripheral neuropathy, so the completion rate was not high at 81.2%. In preoperative chemotherapy studies, completion rates for CAPOX/FOLFOX+Bmab ranged from 72%–97%,^5^, ^6^, ^20^ while completion rates for CAPOX/FOLFOX or FOLFOXIRI were 91%–97%.^19^, ^23^, ^24^ The results raised concerns about the safety of concomitant use of molecular‐targeted drugs, and considering the intestinal perforation in this study, bevacizumab should not be administered when surgery is a prerequisite.

On the other hand, the additive effect of bevacizumab in prolonging survival for unresectable patients was obvious,^25^ and the SOX+Bmab regimen used in this study was comparable to FOLFOX+Bmab,^26^ not to mention that for SOX, higher response rates have been reported compared to CAPOX.^27^ Therefore, now that upfront chemotherapy for unresectable cases has become the standard,^28^ the role of surgical treatment for conversion cases is important. It is known that anastomotic leakage from rectal surgery after bevacizumab administration is high,^5^ but this study is significant because it suggests that anastomotic leakage was less likely to occur if a diverting stoma was created in patients with anastomosis.

Other findings of this study include that when MRI shows a significant response to NAC, local control could be achieved with surgical treatment that avoids radiotherapy, and that cure could be almost expected. One in three patients with NAC for high‐risk LARC achieved ycT0‐3MRF‐LLN‐, with no local recurrence, and was possible for most sphincter‐sparing surgery. Although nonoperative management (NOM) with additional RT for these patients is expected, as shown in this study, high‐risk LARC often requires stoma creation due to stenosis, and NOM would be difficult to achieve in such patients. Furthermore, it is still not clear whether RT or surgery provides a better quality of life, as radiation alone can induce bowel and sexual dysfunction. The development of modalities that can predict successful cases of NAC or RT is necessary to ensure safe and maximized efficacy in tailored strategies that also include NOM.

There is no clear evidence whether LLND, which has been reported to be useful in Japan,^29^ or RT are effective for lateral local control, but an RCT is currently being conducted and the results are awaited (NCT03587480). However, as observed in this study, bilateral LLND is sometimes not performed in cases where it is planned, and preoperative treatment is considered essential when LLND is deemed necessary. In fact, local recurrence in bilateral LLND alone in cases with cLLN+ (≥5 mm in short axis diameter) was very high at 21.4%.^30^ On the other hand, the completion rate of short‐term RT is almost 100%, and a total neoadjuvant therapy (TNT) strategy combining RT with NAC is expected, as its oncological benefit has recently been demonstrated.^31^ Currently, selective LLND after TNT for patients with cLLN+ on MRI is considered a promising strategy that maximizes safety and efficacy, with lateral local recurrence reported to be as low as 3%–4%.^32^ On the other hand, the local recurrence rate of selective LLND after RT for patients with cLLN+ in Japan and Korea was reported to be 3%–5%, and the pathologic LLN+ rate was 24%–51%.^33^, ^34^, ^35^ The pathologic LLN+ rate after NAC for cLLN+ cases in this study was 22%, but the local recurrence rate was as high as 41% even if extended surgeries were performed. Although RT may be necessary for cLLN+ cases, there were no local recurrences in MRF‐ and cLLN‐ or good responders that were ycT0‐3MRF‐LLN‐ after NAC + LLND. This suggested selective LLND after NAC may be sufficient for such cases, but the results were based on a small number of cases and await further investigation.

There are several limitations to this study. Although this is a multicenter study, the number of cases is small, and the true factor contributing to cure may not be due to NAC because it is a single‐arm, non‐comparative, controlled study. Since we did not evaluate postoperative bowel dysfunction in sphincter function‐preserving surgery, it is unclear whether avoidance of RT in this study actually reduced bowel dysfunction. Since most of the cT4b cases were levator ani muscles and there were no pT4b cases, it is possible that obvious urogenital invasion cases were not included. The adjuvant S‐1 monotherapy for high‐risk LARC may have been inadequate in cases of poor response to NAC. TNT would be a reasonable strategy for cases of poor response to NAC because extended surgery was often inevitable and low compliance of adjuvant 5‐FU + oxaliplatin therapy was expected in such cases. Despite these limitations, this study suggests that NAC followed by surgery for high‐risk LARC is a strategy worth considering as an option in future tailored treatment.

## 5 CONCLUSION

There was a clinically significant effect of preoperative SOX+Bmab in about a half of high‐risk LARC patients. If NAC proves inadequately effective, RT should be added for high‐risk LARC, even if extended surgery is intended.

## AUTHOR CONTRIBUTIONS

TM, HM, and YS contributed to the conception and design of the study. TM, HM, YS, TK, TH, YN, and HF performed the acquisition, analysis, or interpretation of data for the study. TM drafted the manuscript. HM, YS, TK, TH, YN, HF, and KH contributed to the review and/or critical revision of the article.

## FUNDING INFORMATION

The authors have no funding to declare.

## CONFLICT OF INTEREST STATEMENT

Author KH is an editorial member of *Annals of Gastroenterological Surgery*.

## ETHICS STATEMENTS

Approval of the research protocol: This study was conducted with the approval of Hirosaki University General Certified Review Board.

Registry and the Registration No. of the study/trial: The Japan Registry of Clinical Trials (jRCTs021180023).

Informed consent: All participating hospitals also gave their consent to conduct this study. Written informed consent was obtained from each patient before enrollment.

Animal studies: NA.

## References

[1] Smith JD , Nash GM , Weiser MR , Temple LK , Guillem JG , Paty PB . Multivisceral resections for rectal cancer. Br J Surg. 2012;99(8):1137–1143. 22696063 10.1002/bjs.8820

[2] Akiyoshi T , Watanabe T , Miyata S , Kotake K , Muto T , Sugihara K , et al. Results of a Japanese nationwide multi‐institutional study on lateral pelvic lymph node metastasis in low rectal cancer: is it regional or distant disease? Ann Surg. 2012;255(6):1129–1134. 22549752 10.1097/SLA.0b013e3182565d9d

[3] Takemasa I , Hamabe A , Miyo M , Akizuki E , Okuya K . Essential updates 2020/2021: advancing precision medicine for comprehensive rectal cancer treatment. Ann Gastroenterol Surg. 2023;7(2):198–215. 36998300 10.1002/ags3.12646 PMC10043777

[4] Pollack J , Holm T , Cedermark B , Holmstrom B , Mellgren A . Long‐term effect of preoperative radiation therapy on anorectal function. Dis Colon Rectum. 2006;49(3):345–352. 16532369 10.1007/s10350-005-0296-1

[5] Uehara K , Hiramatsu K , Maeda A , Sakamoto E , Inoue M , Kobayashi S , et al. Neoadjuvant oxaliplatin and capecitabine and bevacizumab without radiotherapy for poor‐risk rectal cancer: N‐SOG 03 phase II trial. Jpn J Clin Oncol. 2013;43(10):964–971. 23935207 10.1093/jjco/hyt115

[6] Hasegawa J , Nishimura J , Mizushima T , Miyake Y , Kim HM , Takemoto H , et al. Neoadjuvant capecitabine and oxaliplatin (XELOX) combined with bevacizumab for high‐risk localized rectal cancer. Cancer Chemother Pharmacol. 2014;73(5):1079–1087. 24595805 10.1007/s00280-014-2417-9

[7] Patel UB , Brown G , Machado I , Santos‐Cores J , Pericay C , Ballesteros E , et al. MRI assessment and outcomes in patients receiving neoadjuvant chemotherapy only for primary rectal cancer: long‐term results from the GEMCAD 0801 trial. Ann Oncol. 2017;28(2):344–353. 28426108 10.1093/annonc/mdw616

[8] Brown G , Daniels IR , Richardson C , Revell P , Peppercorn D , Bourne M . Techniques and trouble‐shooting in high spatial resolution thin slice MRI for rectal cancer. Br J Radiol. 2005;78(927):245–251. 15730990 10.1259/bjr/33540239

[9] Taylor FG , Swift RI , Blomqvist L , Brown G . A systematic approach to the interpretation of preoperative staging MRI for rectal cancer. AJR Am J Roentgenol. 2008;191(6):1827–1835. 19020255 10.2214/AJR.08.1004

[10] Ogawa S , Hida J , Ike H , Kinugasa T , Ota M , Shinto E , et al. The important risk factor for lateral pelvic lymph node metastasis of lower rectal cancer is node‐positive status on magnetic resonance imaging: study of the lymph node Committee of Japanese Society for cancer of the colon and Rectum. Int J Colorectal Dis. 2016;31(10):1719–1728. 27576475 10.1007/s00384-016-2641-3

[11] Patel UB , Blomqvist LK , Taylor F , George C , Guthrie A , Bees N , et al. MRI after treatment of locally advanced rectal cancer: how to report tumor response—the MERCURY experience. AJR Am J Roentgenol. 2012;199(4):W486–W495. 22997398 10.2214/AJR.11.8210

[12] Eisenhauer EA , Therasse P , Bogaerts J , Schwartz LH , Sargent D , Ford R , et al. New response evaluation criteria in solid tumours: revised RECIST guideline (version 1.1). Eur J Cancer. 2009;45(2):228–247. 19097774 10.1016/j.ejca.2008.10.026

[13] Hashiguchi Y , Muro K , Saito Y , Ito Y , Ajioka Y , Hamaguchi T , et al. Japanese Society for Cancer of the colon and Rectum (JSCCR) guidelines 2019 for the treatment of colorectal cancer. Int J Clin Oncol. 2020;25(1):1–42. 31203527 10.1007/s10147-019-01485-z PMC6946738

[14] Dindo D , Demartines N , Clavien PA . Classification of surgical complications: a new proposal with evaluation in a cohort of 6336 patients and results of a survey. Ann Surg. 2004;240(2):205–213. 15273542 10.1097/01.sla.0000133083.54934.ae PMC1360123

[15] Glynne‐Jones R , Counsell N , Quirke P , Mortensen N , Maraveyas A , Meadows HM , et al. Chronicle: results of a randomised phase III trial in locally advanced rectal cancer after neoadjuvant chemoradiation randomising postoperative adjuvant capecitabine plus oxaliplatin (XELOX) versus control. Ann Oncol. 2014;25(7):1356–1362. 24718885 10.1093/annonc/mdu147

[16] Oki E , Murata A , Yoshida K , Maeda K , Ikejiri K , Munemoto Y , et al. A randomized phase III trial comparing S‐1 versus UFT as adjuvant chemotherapy for stage II/III rectal cancer (JFMC35‐C1: ACTS‐RC). Ann Oncol. 2016;27(7):1266–1272. 27056996 10.1093/annonc/mdw162 PMC4922318

[17] Kanda Y . Investigation of the freely available easy‐to‐use software 'EZR' for medical statistics. Bone Marrow Transplant. 2013;48(3):452–458. 23208313 10.1038/bmt.2012.244 PMC3590441

[18] Deng Y , Chi P , Lan P , Wang L , Chen W , Cui L , et al. Neoadjuvant modified FOLFOX6 with or without radiation versus fluorouracil plus radiation for locally advanced rectal cancer: final results of the Chinese FOWARC trial. J Clin Oncol. 2019;37(34):3223–3233. 31557064 10.1200/JCO.18.02309 PMC6881102

[19] Conroy T , Bosset JF , Etienne PL , Rio E , Francois E , Mesgouez‐Nebout N , et al. Neoadjuvant chemotherapy with FOLFIRINOX and preoperative chemoradiotherapy for patients with locally advanced rectal cancer (UNICANCER‐PRODIGE 23): a multicentre, randomised, open‐label, phase 3 trial. Lancet Oncol. 2021;22(5):702–715. 33862000 10.1016/S1470-2045(21)00079-6

[20] Konishi T , Shinozaki E , Murofushi K , Taguchi S , Fukunaga Y , Nagayama S , et al. Phase II trial of neoadjuvant chemotherapy, Chemoradiotherapy, and laparoscopic surgery with selective lateral node dissection for poor‐risk low rectal cancer. Ann Surg Oncol. 2019;26(8):2507–2513. 30963400 10.1245/s10434-019-07342-7

[21] Rouanet P , Rullier E , Lelong B , Maingon P , Tuech JJ , Pezet D , et al. Tailored strategy for locally advanced rectal carcinoma (GRECCAR 4): long‐term results from a multicenter, randomized, open‐label. Phase II Trial Dis Colon Rectum. 2022;65(8):986–995. 34759247 10.1097/DCR.0000000000002153

[22] Chua YJ , Barbachano Y , Cunningham D , Oates JR , Brown G , Wotherspoon A , et al. Neoadjuvant capecitabine and oxaliplatin before chemoradiotherapy and total mesorectal excision in MRI‐defined poor‐risk rectal cancer: a phase 2 trial. Lancet Oncol. 2010;11(3):241–248. 20106720 10.1016/S1470-2045(09)70381-X

[23] Fokas E , Allgauer M , Polat B , Klautke G , Grabenbauer GG , Fietkau R , et al. Randomized phase II trial of Chemoradiotherapy plus induction or consolidation chemotherapy as Total neoadjuvant therapy for locally advanced rectal cancer: CAO/ARO/AIO‐12. J Clin Oncol. 2019;37(34):3212–3222. 31150315 10.1200/JCO.19.00308

[24] Fernandez‐Martos C , Garcia‐Albeniz X , Pericay C , Maurel J , Aparicio J , Montagut C , et al. Chemoradiation, surgery and adjuvant chemotherapy versus induction chemotherapy followed by chemoradiation and surgery: long‐term results of the Spanish GCR‐3 phase II randomized trialdagger. Ann Oncol. 2015;26(8):1722–1728. 25957330 10.1093/annonc/mdv223

[25] Saltz LB , Clarke S , Diaz‐Rubio E , Scheithauer W , Figer A , Wong R , et al. Bevacizumab in combination with oxaliplatin‐based chemotherapy as first‐line therapy in metastatic colorectal cancer: a randomized phase III study. J Clin Oncol. 2008;26(12):2013–2019. 18421054 10.1200/JCO.2007.14.9930

[26] Yamada Y , Takahari D , Matsumoto H , Baba H , Nakamura M , Yoshida K , et al. Leucovorin, fluorouracil, and oxaliplatin plus bevacizumab versus S‐1 and oxaliplatin plus bevacizumab in patients with metastatic colorectal cancer (SOFT): an open‐label, non‐inferiority, randomised phase 3 trial. Lancet Oncol. 2013;14(13):1278–1286. 24225157 10.1016/S1470-2045(13)70490-X

[27] Hong YS , Park YS , Lim HY , Lee J , Kim TW , Kim KP , et al. S‐1 plus oxaliplatin versus capecitabine plus oxaliplatin for first‐line treatment of patients with metastatic colorectal cancer: a randomised, non‐inferiority phase 3 trial. Lancet Oncol. 2012;13(11):1125–1132. 23062232 10.1016/S1470-2045(12)70363-7

[28] Kanemitsu Y , Shitara K , Mizusawa J , Hamaguchi T , Shida D , Komori K , et al. Primary tumor resection plus chemotherapy versus chemotherapy alone for colorectal cancer patients with asymptomatic, synchronous Unresectable metastases (JCOG1007; iPACS): a randomized clinical trial. J Clin Oncol. 2021;39(10):1098–1107. 33560877 10.1200/JCO.20.02447 PMC8078424

[29] Fujita S , Mizusawa J , Kanemitsu Y , Ito M , Kinugasa Y , Komori K , et al. Mesorectal excision with or without lateral lymph node dissection for clinical stage II/III lower rectal cancer (JCOG0212): a multicenter, randomized controlled. Noninferiority Trial Ann Surg. 2017;266(2):201–207. 28288057 10.1097/SLA.0000000000002212

[30] Komori K , Fujita S , Mizusawa J , Kanemitsu Y , Ito M , Shiomi A , et al. Predictive factors of pathological lateral pelvic lymph node metastasis in patients without clinical lateral pelvic lymph node metastasis (clinical stage II/III): the analysis of data from the clinical trial (JCOG0212). Eur J Surg Oncol. 2019;45(3):336–340. 30477950 10.1016/j.ejso.2018.11.016

[31] Bahadoer RR , Dijkstra EA , van Etten B , Marijnen CAM , Putter H , Kranenbarg EM , et al. Short‐course radiotherapy followed by chemotherapy before total mesorectal excision (TME) versus preoperative chemoradiotherapy, TME, and optional adjuvant chemotherapy in locally advanced rectal cancer (RAPIDO): a randomised, open‐label, phase 3 trial. Lancet Oncol. 2021;22(1):29–42. 33301740 10.1016/S1470-2045(20)30555-6

[32] Peacock O , Manisundaram N , Dibrito SR , Kim Y , Hu CY , Bednarski BK , et al. Magnetic resonance imaging directed surgical decision making for lateral pelvic lymph node dissection in rectal cancer after Total neoadjuvant therapy (TNT). Ann Surg. 2022;276(4):654–664. 35837891 10.1097/SLA.0000000000005589 PMC9463102

[33] Ogura A , Akiyoshi T , Nagasaki T , Konishi T , Fujimoto Y , Nagayama S , et al. Feasibility of laparoscopic Total Mesorectal excision with extended lateral pelvic lymph node dissection for advanced lower rectal cancer after preoperative Chemoradiotherapy. World J Surg. 2017;41(3):868–875. 27730352 10.1007/s00268-016-3762-0

[34] Ishihara S , Kawai K , Tanaka T , Kiyomatsu T , Hata K , Nozawa H , et al. Oncological outcomes of lateral pelvic lymph node metastasis in rectal cancer treated with preoperative Chemoradiotherapy. Dis Colon Rectum. 2017;60(5):469–476. 28383446 10.1097/DCR.0000000000000752

[35] Kim MJ , Chang GJ , Lim HK , Song MK , Park SC , Sohn DK , et al. Oncological impact of lateral lymph node dissection after preoperative Chemoradiotherapy in patients with rectal cancer. Ann Surg Oncol. 2020;27(9):3525–3533. 32385767 10.1245/s10434-020-08481-y

